# Decreased Gray-Matter Volume in Insular Cortex as a Correlate of Singers’ Enhanced Sensorimotor Control of Vocal Production

**DOI:** 10.3389/fnins.2019.00815

**Published:** 2019-08-02

**Authors:** Wenda Wang, Lirao Wei, Na Chen, Jeffery A. Jones, Gaolang Gong, Hanjun Liu

**Affiliations:** ^1^Department of Rehabilitation Medicine, The First Affiliated Hospital, Sun Yat-sen University, Guangzhou, China; ^2^Department of Rehabilitation, Guangzhou Women and Children’s Medical Center, Guangzhou Medical University, Guangzhou, China; ^3^Department of Music, Guangdong University of Education, Guangzhou, China; ^4^Department of Rehabilitation, Zhujiang Hospital, Southern Medical University, Guangzhou, China; ^5^Psychology Department and Laurier Centre for Cognitive Neuroscience, Wilfrid Laurier University, Waterloo, ON, Canada; ^6^State Key Laboratory of Cognitive Neuroscience and Learning and IDG/McGovern Institute for Brain Research, Beijing Normal University, Beijing, China; ^7^Guangdong Provincial Key Laboratory of Brain Function and Disease, Zhongshan School of Medicine, Sun Yat-sen University, Guangzhou, China

**Keywords:** auditory feedback, speech motor control, voxel-based morphology, insula, singing

## Abstract

Accumulating evidence has shown enhanced sensorimotor control of vocal production as a consequence of extensive singing experience. The neural basis of this ability, however, is poorly understood. Given that the insula mediates motor aspects of vocal production, the present study investigated structural plasticity in insula induced by singing experience and its link to auditory feedback control of vocal production. Voxel-based morphometry (VBM) was used to examine the differences in gray matter (GM) volume in the insula of 21 singers and 21 non-singers. An auditory feedback perturbation paradigm was used to examine the differences in auditory-motor control of vocal production between singers and non-singers. Both groups vocalized sustained vowels while hearing their voice unexpectedly pitch-shifted −50 or −200 cents (200 ms duration). VBM analyses showed that singers exhibited significantly lower GM volumes in the bilateral insula than non-singers. When exposed to pitch perturbations in voice auditory feedback, singers involuntarily compensated for pitch perturbations in voice auditory feedback to a significantly lesser degree than non-singers. Moreover, across the two sizes of pitch perturbations, the magnitudes of vocal compensations were positively correlated with the total regional GM volumes in the bilateral insula. These results indicate that extensive singing training leads to decreased GM volumes in insula and suggest that morphometric plasticity in insula contributes to the enhanced sensorimotor control of vocal production observed in singers.

## Introduction

The goal of speech motor control is intelligible speech sounds. These sounds, perceived as auditory feedback, provide critical information that allows the brain to detect errors in vocal output and initiate motor commands that correct for them (Guenther, 2006; Houde and Chang, 2015). Speakers have been consistently shown to compensate for perturbations heard in their voice fundamental frequency (*F*_0_), intensity, and formant frequency (Burnett et al., 1998; Houde and Jordan, 1998; Jones and Munhall, 2005; Bauer et al., 2006; Purcell and Munhall, 2006; Liu and Larson, 2007). This compensatory process engages a large complex neural network of sensory, motor, and cognitive systems (Tourville et al., 2008; Parkinson et al., 2012; Chang et al., 2013; Kort et al., 2014; Behroozmand et al., 2015, 2017). Our understanding of sensorimotor integration for voice control, however, is still far from clear.

A growing body of literature has shown that, across participants, the ability to compensate for perturbations in voice auditory feedback is not equal, but rather varies as a function of expertise like singing. For example, a series of singing studies found that, when auditory feedback was perturbed in pitch during syllable singing, singers suppressed their vocal compensations for pitch perturbations to a lesser degree and were even able to completely ignore large pitch perturbations (i.e., produced almost no vocal compensation) compared to non-singers (Zarate and Zatorre, 2005, 2008; Zarate et al., 2010). This behavioral difference between the two groups was accompanied by distinct neural networks: singers showed increased activity in the inferior parietal lobule (IPL), superior temporal gyrus (STG), superior temporal sulcus (STS), and insula while non-singers recruited the anterior cingulate cortex (ACC), premotor cortex (PMC), and supramarginal gyrus (SMG) (Zarate and Zatorre, 2005, 2008). Music experience can also influence the reaction time that participants correct for voice pitch feedback errors, as reflected by longer reaction time for highly skilled singers than for moderately skilled singers (Grell et al., 2009). In one sensorimotor adaptation study by Jones and Keough (2008), singers not only produced smaller vocal compensations than non-singers during the pitch perturbation phase but also exhibited a larger aftereffect as reflected by higher *F*_0_ values when auditory feedback returned normal compared to the baseline phase (i.e., no perturbation). Similarly, Kleber et al. (2017) found singers’ pitch matching accuracy to be significantly more preserved than non-singers when their auditory feedback was masked by noise. These results suggest that singing experience may lead to decreased reliance on auditory feedback and that instead, trained singers may rely relatively more on feedforward control mechanisms or the “acquired neuromuscular memory of pitch” (Murbe et al., 2004) to produce the vocal targets with increased precision.

Note that the aforementioned studies of speech motor control instructed musically trained participants to sing the syllables, whereas other studies that involved non-musicians typically employed speaking tasks (i.e., vocalizing the vowel sounds) (Burnett et al., 1998; Liu and Larson, 2007; Liu et al., 2011; Scheerer et al., 2013; Kort et al., 2014; Behroozmand et al., 2015). Despite the fundamental similarity in the way pitch is used for singing and speaking, singing requires more accurate encoding of pitch information and a higher level of vocal motor control than does speaking (Zatorre and Baum, 2012), which leads to the additional recruitment of right-hemisphere brain regions that include the STG and inferior frontal gyrus (IFG) as well as the insula (Riecker et al., 2000; Ozdemir et al., 2006). One behavioral study showed that singing the syllables leads to larger vocal compensations for pitch perturbations than speaking the syllables in non-musicians (Natke et al., 2003). It should be noted that the previously reported vocal responses produced by singers were measured 1,900–3,000 ms after the onset of the 3-s-long pitch perturbation (Zarate and Zatorre, 2008; Zarate et al., 2010). It has been suggested that these relatively late responses are voluntary and reflect a conscious strategy to oppose perceived changes in voice auditory feedback (Burnett et al., 1998; Hain et al., 2000). In contrast, other studies that involved non-musicians measured the vocal responses 50–400 ms after the onset of a pitch perturbation that was 200–400 ms long (Chen et al., 2007; Liu and Larson, 2007; Parkinson et al., 2012; Scheerer et al., 2013; Kort et al., 2014). These early responses are reflex-like or involuntary and are unlikely to be consciously modified. Therefore, singers’ ability to suppress compensatory adjustments of their vocal motor behaviors may be influenced by the specificity of the task demands (i.e., singing vs. speaking) and the nature of the vocal responses (i.e., voluntary vs. involuntary). This idea is supported by one study by Behroozmand et al. (2014) who showed that musicians with absolute pitch (AP) and relative pitch (RP) did not suppress their vocal responses, but instead compensated for the pitch perturbations to a relatively larger degree than non-musicians when they vocalized the vowel sounds. However, because Behroozmand et al. (2014) included both singers and instrumentalists in their AP and RP musician groups, it remains unclear how the integration of auditory feedback into ongoing voice control is modulated by singing experience.

Despite considerable research on experience-dependent functional changes in the cortical representations of sensorimotor integration for speech (Riecker et al., 2005; Zarate and Zatorre, 2005, 2008; Ozdemir et al., 2006; Kleber et al., 2010; Zarate et al., 2010), structural plasticity as a function of singing expertise and the assessment of its relationship with speech motor control have rarely been investigated. Numerous studies have shown that becoming a proficient instrumentalist leads to increased gray matter (GM) volume and cortical thickness in the auditory- and motor-related regions as well as reorganization of white matter (WM) (Schneider et al., 2002; Gaser and Schlaug, 2003; Bengtsson et al., 2005; Bermudez et al., 2009; Steele et al., 2013; Groussard et al., 2014). In contrast, only a few studies have investigated the structural plasticity induced by singing experience. In one diffusion tensor imaging (DTI) study by Halwani et al. (2011), both trained singers and instrumentalists exhibited larger WM tract volumes than non-musicians in the right arcuate fasciculus (AF), which connects fronto-temporal, sensorimotor, and inferior parietal regions (Glasser and Rilling, 2008). In a more recent voxel-based morphometry (VBM) study by Kleber et al. (2016), singers exhibited larger GM volumes in the right primary and secondary somatosensory cortices (S1 and S2), rostral SMG, and primary auditory cortex (A1), regions that have been shown to be active in compensating for perturbed voice *F*_0_ during speaking (Tourville et al., 2008; Chang et al., 2013; Behroozmand et al., 2015; Kort et al., 2016). Therefore, it is reasonable to hypothesize that long-term auditory-vocal training leads to structural changes in brain regions that are functionally relevant for sensorimotor control of speech production, and that the assessment of their relationship may provide a window into the structural basis of speech motor control as a function of singing expertise.

The present VBM study investigated the neuroanatomical correlates of auditory feedback control of vocal pitch regulation in singers with the intention of (1) revealing whether GM volume differences in an *a priori* region of interest (ROI) would exist between singers and non-singers, and (2) examining the relationship between GM volume in the selected ROI and participants’ vocal compensations for pitch perturbations in auditory feedback. The ROI selected for the present study was the insular cortex bilaterally. The insula is a complex structure that has a wide array of cortical connections with the frontal (e.g., PMC), temporal (e.g., STG, STS), and parietal (e.g., IPL) regions (Shelley and Trimble, 2004; Ghaziri et al., 2017). These widespread connections between the insula and other brain regions provide a role for the insula in a variety of sensorimotor integration and cognitive functions, including speech/language processing (Dronkers, 1996), central audition (Bamiou et al., 2006), body awareness (Craig, 2009), salience detection (Critchley et al., 2004), and affective processes (Deen et al., 2011; Uddin et al., 2014). More specifically, evidence from lesion and neuroimaging studies has demonstrated the involvement of the insula in the motor control of speech production. For example, lesions to the insula can lead to deficits in speech articulation and motor planning, such as apraxia of speech, reduced fluency, and impairments with articulatory movement (Dronkers, 1996; Bates et al., 2003; Ackermann and Riecker, 2010). Neuroimaging studies of healthy populations have identified increased activation in the insula during the production of compensatory vocal responses to pitch perturbations during singing (Zarate and Zatorre, 2008; Zarate et al., 2010) and speaking (Toyomura et al., 2007; Behroozmand et al., 2015; Kort et al., 2016). Moreover, Kleber et al. (2013) reported that, when asked to maintain pitch-matching performance after anesthesia of the vocal tracts, singers exhibited decreased activity in the right anterior insula and decreased connectivity between the insula and the auditory and somatosensory regions. In a subsequent study by Kleber et al. (2017), singers exhibited increased activity in the right anterior insula and increased connectivity between the insula and the SMG when they sang in the absence of auditory feedback. These findings suggest that the insula serves as a critical hub for the coordination of large-scale brain networks involved in integrating sensory, somatosensory, and motor information for speech motor control (Zarate and Zatorre, 2008).

Based on previous research that has shown structural neuroplasticity as a function of singing experience and the contribution of the insula to speech motor control, we predicted that singers and non-singers would significantly differ in GM volume in the selected ROI (i.e., bilateral insular cortex) and auditory feedback control of vocal pitch production. Furthermore, we predicted that a significant correlation would exist between GM volume in the insula and participants’ ability to compensate for vocal pitch perturbations. The results confirmed our hypotheses; we observed reduced regional GM volume in the bilateral insula and decreased involuntary vocal compensations for pitch perturbations in singers and positive correlations between GM volumes in those regions and the magnitudes of vocal compensations.

## Materials and Methods

### Subjects

A total of 42 college students participated in the experiment. A group of 21 female classically trained singers [19–29 years, mean = 24.09, standard deviation (*SD*) = 2.19], consisting of 4 undergraduate students and 17 graduate students, were recruited from the Department of Music at South China Normal University. The participants took their formal singing lessons from the average age of 12 ± 5 years (range: 4–21 years) and studied for an average of 12 ± 5 years (range: 5–20 years). Some of the trained singers also played the piano. In addition, all singers reported that they did not possess AP. Twenty-one female college students (18–27 years, mean = 23.17, *SD* = 2.65) without previous vocal training or instrumental playing experience were recruited from Sun Yat-sen University (4 undergraduate and 17 graduate students) and assigned to the non-singers group. The two groups were matched in age (*t* = 1.139, *p* = 0.268), gender, and education. Based on self-report measures of alcohol and tobacco use, all participants were classified as non-drinker (0 units of alcohol per week) and non-smokers (0 cigarettes per day). All participants were right-handed and native Mandarin speakers. They reported no history of speech, language, hearing, and neurological disorders. All participants passed a binaural hearing screening at the threshold of 25 dB HL for pure tone frequencies of 0.5–4 kHz. They received monetary compensation for their participation and gave written informed consent in compliance with a research protocol approved by the Institution Review Board of The First Affiliated Hospital at Sun Yat-sen University of China.

### Structural MRI Acquisition

Structural MRI data were acquired on a Siemens Magnetom 3T Trio Tim MRI scanner (Erlangen, Germany) located at South China Normal University. During acquisition, all participants were required to lie still and stay awake with their eyes closed. The high-resolution anatomical images were acquired using T1-weighted 3D Magnetization Prepared Rapid Gradient Echo (MPRAGE) sequence with the following parameters: TR (repetition time) = 2,300 ms; TE (echo time) = 3.24 ms; flip angle = 9°; FOV (field of view) = 256 × 256 mm^2^; slices thickness = 1 mm; and voxel size = 1 × 1 × 1 mm.

### Vocal Data Acquisition

After the MRI data acquisition, both singers and non-singers participated in a vocal production experiment using the frequency-altered feedback (FAF) paradigm. They were instructed to vocalize the vowel sound /u/ for approximately 5–6 s and to speak at their comfortable pitch and loudness level. During each vocalization, participants’ voices were shifted down in pitch by 50 or 200 cents (100 cents equals one semitone) five times. The duration of each pitch perturbation was fixed at 200 ms. The two sizes of pitch perturbations were presented pseudorandomly across all participants; the initial pitch perturbation occurred with a delay of 500–1,000 ms relative to the vocal onset, and the succeeding stimuli were presented with an inter-stimulus interval of 700–900 ms. Each participant was required to take a break of 2–3 s between successive vocalizations and produced 40 consecutive vocalizations. A total of 200 trials were thus collected, including 100 trials for the −50 cents condition and 100 trials for the −200 cents condition.

Throughout the experiment, the vocal data were collected from participants while they sat in a sound-treated booth. The voice feedback was calibrated to be 10 dB SPL higher than that of participant’s vocal output using a Zwisklocki coupler and a Brüel & Kjaer sound level meter (model 2250) to reduce the influence of the air-born and bone-conducted voice feedback (Larson et al., 2008; Patel et al., 2014). During the experiment, the voice signals were recorded via a dynamic microphone (DM2200, Takstar Inc.), amplified with a MOTU Ultralite Mk3 Firewire audio interface, and pitch-shifted by an Eventide Eclipse Harmonizer controlled by a Max/MSP software program (v.5.0 by Cycling 74). The pitch-shifted voice signals were then amplified by an ICON NeoAmp headphone amplifier and presented to participants through insert earphones (ER1, Etymotic Research Inc.). Transistor–transistor logic (TTL) pulses were generated by the Max/MSP software program to mark each pitch shift event for averaging the vocal trials. The acoustic data and the TTL pulses were recorded at a sampling frequency of 10 kHz by a PowerLab A/D converter (model ML880, AD Instruments) using LabChart software (v.7.0 by AD Instruments). Note that the scalp-recorded electroencephalography (EEG) data were also recorded using a 64-electrode Geodesic Sensor Net through a Net Amps 300 amplifier (Electrical Geodesics Inc., Eugene, OR, United States), but the ERP results are not reported here.

### Data Analysis

#### Vocal Data Analysis

Compensatory vocal responses to pitch perturbations were measured using IGOR PRO software (v.6.0 by Wavemetrics Inc.) using the event-related averaging technique (Liu and Larson, 2007; Larson et al., 2008). The voice *F*_0_ contours were extracted using Praat software (Boersma, 2001) and converted to the cent scale with the following formula: cents = 100 × (12 × log_2_(*F*_0_/reference)) [reference = 195.997 Hz (G3 note)]. The voice contours were then segmented into trials with a window of 200 ms before and 700 ms after the perturbation onset. A visual inspection was performed to remove those trials that were contaminated by errors in vocal production or signal processing. Those artifact-free trials that opposed the direction of pitch perturbations were averaged and baseline-corrected to generate an overall compensatory vocal response for each condition (Li et al., 2013). The magnitude and latency of a vocal response were defined as the *F*_0_ value in cents and time in ms when the voice *F*_0_ contours reached their maximum values, respectively. We chose a time window of 50–400 ms after the perturbation onset to detect the prominent peaks of the involuntary vocal responses based on the present and previous studies (Burnett et al., 1998; Behroozmand et al., 2014; Tang et al., 2018).

### MRI Data Processing

Structural MRI image data were processed using the Statistic Parametric Mapping software (SPM 12^1^). The CAT12 toolbox^2^ implemented in SPM 12 was used for VBM analysis (Ashburner and Friston, 2000). First, all T1-weighted images were segmented into GM, WM, and cerebral spinal fluid (CSF) for the calculation of the overall tissue volume (GM, WM, and CSF) and total intracranial volume (TIV) in the native space. An internal GM threshold of 0.2 was used to exclude those artifacts on the gray-white-matter border. All of the segmented tissues were then registered to the standard Montreal Neurological Institute template in SPM12 using the affine registration algorithm. The Diffeomorphic Anatomical Registration Through Exponentiated Lie Algebra (DARTEL) toolbox was used to refine the inter-subject registration of all participants’ GM and WM. A statistical quality check procedure was performed using the CAT12 toolbox to assess the homogeneity of the GM tissues when the preprocessing pipeline was completed. Finally, normalized GM tissue segments for each participant were smoothed with an 8-mm full width at half maximum (FWHM) Gaussian filter.

### Statistical Analysis

The magnitudes and latencies of vocal compensations for pitch perturbations were subjected to repeated-measures analyses of variance (RM-ANOVAs). Group (singers vs. non-singers) was chosen as a between-subject factor, while stimulus magnitude (−50 cents vs. −200 cents) was chosen as a within-subject factor. Significant higher-order interactions between two variables led to subsidiary RM-ANOVAs. Probability values were corrected for multiple degrees of freedom when violations of sphericity occurred. The effect size indexed by $\eta_{\text{p}}^{2}$ was calculated to indicate the size of differences across the conditions. An alpha level of *p* < 0.05 was considered to be significant.

Anatomical group differences with respect to GM volume were assessed in *ad hoc* defined ROIs, focusing on the left and right insular cortex. In this ROI analysis, the bilateral insular cortices were selected as masks from the automated anatomical labeling (AAL) template (Tzourio-Mazoyer et al., 2002) using the Data Processing and Analysis of Brain Imaging (DPABI) MATLAB toolbox implemented in SPM 12 (Yan et al., 2016). A voxel-based comparison with independent two-sample *t*-tests was performed to detect differences between singers and non-singers within the mask. The significance of group differences in the ROI was estimated to correct for multiple comparisons using the Gaussian random field (GRF) theory (voxel-level significance: *p* < 0.001; cluster-level significance: *p* < 0.05) with covariates of age and TIV included. We extracted the total GM volumes of each significant cluster within each ROI for each subject using DPABI. The Pearson correlation analysis was performed to assess the relationship between the total GM volumes in each significant cluster and the magnitudes of vocal compensations for −50 and −200 cents perturbations with a significance level of *p* < 0.05 for all statistical analyses.

## Results

### Comparisons of Vocal Responses

**FIGURE 1:**
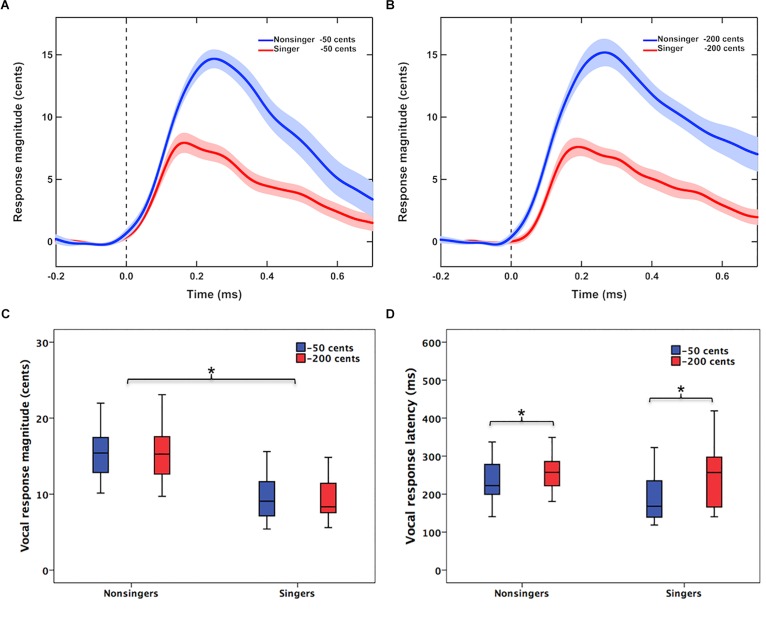
**(A,B)** Grand-averaged voice *F*_0_ contours in response to –50 cents and –200 cents pitch perturbations for singers (red lines) and non-singers (blue lines). The vertical dashed lines indicate the onset of pitch perturbations, and the highlighted areas denote the standard errors of the mean vocal responses. **(C,D)** Box plots that illustrate the magnitudes and latencies of involuntary vocal compensations for –50 cents (blue boxes) and –200 cents (red boxes) pitch perturbations by singers and non-singers. The asterisks indicate significant differences across the conditions. The top and bottom of boxes indicate the third quartile and the first quartile, and the horizontal lines in the middle of the boxes indicate the median.

Figure 1 shows the grand-averaged voice *F*_0_ contours in response to −50 and −200 cents pitch perturbations for singers and non-singers. Regardless of the size of pitch perturbations, singers exhibited smaller involuntary vocal compensations than non-singers (Figures 1A,B). This difference was demonstrated by a significant main effect of group [*F*(1,40) = 36.834, *p* < 0.001, $\eta_{\text{p}}^{2}$ = 0.479] in a two-way RM-ANOVA conducted on the magnitude of vocal compensation; singers produced significantly smaller vocal compensations than non-singers (9.4 ± 2.7 cents vs. 15.8 ± 4.4 cents) (Figure 1C). The main effect of stimulus magnitude [*F*(1,40) = 0.003, *p* = 0.953, $\eta_{\text{p}}^{2}$ < 0.001] (12.6 ± 4.7 cents vs. 12.6 ± 5.1 cents), however, did not reach significance. Neither did the interaction between group and stimulus magnitude [*F*(1,40) = 0.656, *p* = 0.423, $\eta_{\text{p}}^{2}$ = 0.016].

In addition, the latencies of vocal compensations were modulated as a function of stimulus magnitude [*F*(1,40) = 7.156, *p* = 0.011, $\eta_{\text{p}}^{2}$ = 0.152], with faster vocal responses (i.e., smaller peak times) observed for −50 cents perturbations than for −200 cents perturbations (216 ± 70 vs. 252 ± 78 ms) (Figure 1D). Although singers appeared to produce faster vocal responses than non-singers (223 ± 90 vs. 244 ± 57 ms), this difference was not statistically significant [*F*(1,40) = 1.336, *p* = 0.255, $\eta_{\text{p}}^{2}$ = 0.032]. The interaction between group and stimulus magnitude was not significant either [*F*(1,40) = 0.980, *p* = 0.328, $\eta_{\text{p}}^{2}$ = 0.024].

### Comparisons of GM Volumes in Insula

**FIGURE 2:**
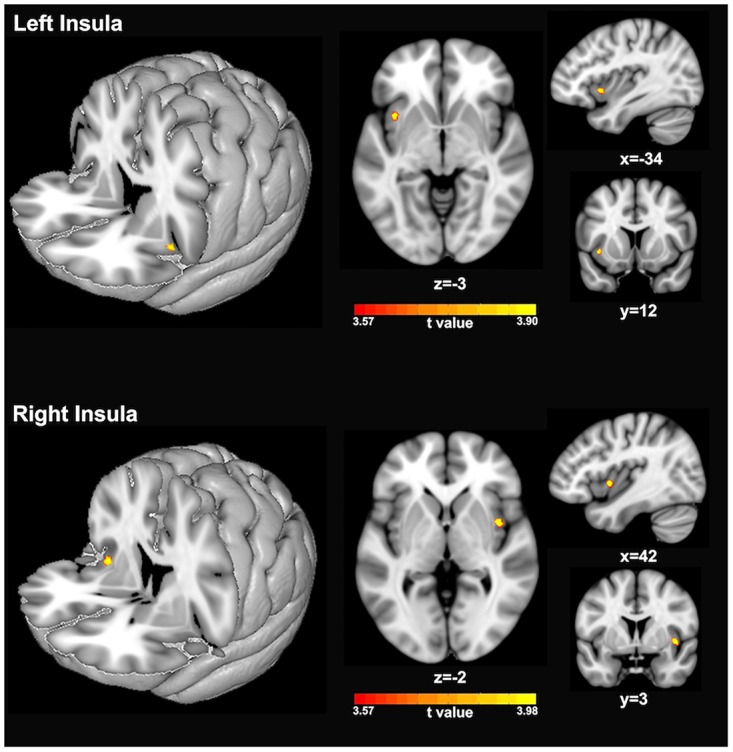
Statistical maps showing significantly lower GM volumes of left insula **(top panel)** and right insula **(bottom panel)** in singers compared to non-singers. The color bars indicate the *t*-values of two-sample *t*-test analyses.

Figure 2 and Table 1 show the voxel-wise group comparison of GM volumes in left and right insula between singers and non-singers. As compared to non-singers, singers exhibited significantly lower GM volumes in the left insula (cluster level GRF corrected, *p* < 0.05; MNI peak coordinate, −34 12 −3) and right insula (cluster level GRF corrected, *p* < 0.05, MIN peak coordinate, 42 3 −2).

**TABLE 1:** Brain regions that showed significantly smaller gray matter volumes in singers compared to non-singers.

	**MNI coordinates**				
**Cluster location**	**x**	**y**	**z**	**Cluster size**	***t*-value**
Left anterior insula	−34	12	−3	34	3.8965
Right posterior insula	42	3	−2	45	3.9792

### The Brain–Behavior Relationship

**FIGURE 3:**
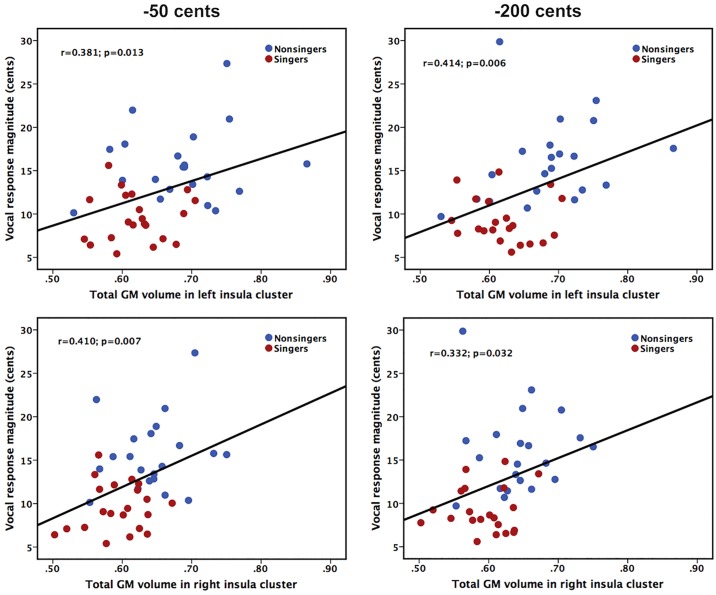
Scatter plots illustrating significant correlations between the total GM volumes of left **(top panel)** and right **(bottom panel)** significant insula clusters and the magnitudes of vocal compensations for –50 cents **(left panel)** and –200 cents **(right panel)** pitch perturbations in the combined cohort of singers and non-singers.

In order to examine whether differences in insula morphology between singers and non-singers contributed to differences in their ability to compensate for voice pitch feedback perturbations, we performed Pearson correlation analyses by correlating the total GM volumes of significant insula clusters with the magnitudes of vocal compensations across the groups. As shown in Figure 3, on the combined cohort of both singers and non-singers, there were significant correlations between insula morphology and auditory-vocal integration. The total GM volume in the significant clusters of the left insula was positively correlated with the magnitude of vocal compensation for −50 cents (*r* = 0.381, *p* = 0.013) and −200 cents (*r* = 0.414, *p* = 0.006) pitch perturbations, respectively. Likewise, the total GM volume in the significant clusters of the right insula was positively correlated with the magnitude of vocal compensation for −50 cents (*r* = 0.410, *p* = 0.007) and −200 cents (*r* = 0.332, *p* = 0.032) pitch perturbations. Therefore, lower GM volumes in the left and right regional insula were predictive of smaller involuntary vocal compensations for pitch perturbations.

## Discussion

The present study investigated structural changes in the insula induced by singing experience and their relationship with auditory feedback control of vocal pitch production. When perceiving pitch perturbations in voice auditory feedback, singers produced involuntary vocal compensations to a significantly lesser degree than non-singers. Interestingly, singers did not completely ignore larger pitch perturbations (i.e., −200 cents) but rather produced similar vocal responses to small pitch perturbations (i.e., −50 cents). VBM analysis revealed morphological changes in insula as a function of singing experience, as reflected by significantly lower GM volumes in the significant clusters of the left and right insula in singers relative to non-singers. Moreover, significant positive correlations were found between the total GM volumes of significant clusters in the insula and the magnitudes of compensatory vocal responses. These findings provide morphometric evidence that reduced GM volume in insula contributes to singers’ ability to suppress involuntary vocal responses to pitch perturbations during speaking as a consequence of singing experience.

### Speech Motor Control in Singers

Previous studies have shown that singers compensate for pitch perturbations to a lesser degree than non-singers in a voluntary manner when they sing the vowel/syllable at a specific note (Zarate and Zatorre, 2005, 2008; Jones and Keough, 2008). Likewise, our behavioral results revealed smaller involuntary vocal responses to pitch perturbations produced by singers compared to non-singers when they were instructed to vocalize the vowel sounds during speaking. In contrast, Behroozmand et al. (2014) reported no significant differences in the magnitudes of involuntary vocal compensations for pitch perturbations between musicians with AP and RP and non-musicians. We speculate that differences between the population samples may be responsible for the inconsistency between our results and those of Behroozmand et al. (2014). For the present study we recruited a homogenous sample of female-only professional singers who did not possess AP, while Behroozmand et al. (2014) study involved both female and male singers and instrumentalists with AP and RP. Future studies need to be conducted to examine the differential effects of vocal and instrument training, as well as sex, on speech motor control.

Note that in the present study singers compensated for pitch perturbations to a lesser degree than non-singers regardless of the size of pitch perturbations, whereas in other studies (Zarate and Zatorre, 2008; Zarate et al., 2010) singers successfully ignored large pitch perturbations (e.g., 200 cents) but failed to ignore small perturbations (e.g., 25 cents). This disparity may be accounted for by methodological differences between the present study and Zarate and colleagues’ work. The present study delivered a number of 200-ms-long pitch perturbations at mid-utterance and measured the vocal responses that began 100–300 ms after the perturbation onset. These early vocal responses are thought to be involuntary and cannot be consciously modulated by singers or non-singers (Munhall et al., 2009; Keough et al., 2013). In the studies by Zarate and Zatorre (2005, 2008) and Zarate et al. (2010), however, the pitch perturbations occurred 1–1.5 s after the vocal onset, lasted until the end of singing, and the vocal responses were measured only for the last second of each 4-s long singing. Their data, therefore, only included late vocal responses, which are subject to voluntary control in a top-down manner (Burnett et al., 1998; Hain et al., 2000; Patel et al., 2014). Thus, it is likely that singers are more successful than non-singers at suppressing vocal compensations for large pitch perturbations that are long enough to allow a voluntary response, but suppressed vocal compensations for both small and large perturbations in singers observed in the present study cannot be consciously modulated because these responses are involuntary in nature.

### Morphometric Changes in the Insula in Singers

Numerous studies have demonstrated that instrumental practice results in increased GM volume and cortical thickness in auditory, motor, and fronto-parietal regions as well as altered WM tracts (Schneider et al., 2002; Gaser and Schlaug, 2003; Bengtsson et al., 2005; Bermudez et al., 2009; Steele et al., 2013; Bailey et al., 2014; Groussard et al., 2014; Schlaug, 2015). For example, compared to non-musicians, instrumental musicians exhibited increased GM volume and cortical thickness in superior temporal regions (Bermudez et al., 2009), and increased brain activity in A1 and GM volume in the anteromedial portion of Heschl’s gyrus (Schneider et al., 2002). Other research has shown a relationship between structural change and musical practice, with longer instrumental practice resulting in greater GM volumes in the left temporal and right frontal cortices, right somatosensory motor areas, and insula (Groussard et al., 2014). With regard to structural plasticity as a function of singing experience, Halwani et al. (2011) found a larger WM tract volume in the right AF in both trained singers and instrumental musicians compared to non-musicians, while Kleber et al. (2016) found increased GM volumes in the right auditory and somatosensory cortices in singers. In the present study, we found significantly lower regional GM volumes in the left and right insula in singers relative to non-singers, providing further evidence in support of structural changes in GM and WM in brain regions that are fundamental to both singing and speaking as a function of singing experience. It is noteworthy that we also chose several other regions that are functionally related to speaking and singing (e.g., ACC, PMC, STG, etc.) as ROIs for VBM analyses but failed to find significant group differences in the GM volumes of those regions after multiple comparison correction, strengthening the role of the insula as an important hub within the speaking/singing network.

Interestingly, despite the previously reported increased GM volume, cortical thickness, or WM tract volume observed in singers, our results showed reduced regional GM volume in the insula in singers. Likewise, several other studies reported that smaller GM volumes were associated with better motor or cognitive performance (Draganski et al., 2006; Hanggi et al., 2010; Duan et al., 2012). Despite the methodological differences, our finding is accordance with a few studies showing negative relations between GM density/cortical thickness and speech learning proficiency (Beal et al., 2007; Dickerson et al., 2008; Rodriguez et al., 2018). For example, higher GM density in the IFG and STG and WM density in the insula were associated with adults who stutter relative to non-stutters (Beal et al., 2007), suggesting a relationship between atypical structural development and deficits in fluent speech production. Better performance on the California Verbal Learning Test was associated with thinner cortex in the paracentral/cingulate sulcus region (Dickerson et al., 2008). More recently, Rodriguez et al. (2018) found that thinner cortical thickness in the left anterior insula was predictive of better discrimination of novel speech sound contrasts in bilinguals, reflecting a more efficiently organized neural network that allows for increased speech learning proficiency. Similar effects of musical experience on the axonal membrane have been found, as reflected by lower fractional anisotropy values in the bilateral corticospinal tract in musicians relative to non-musicians (Schmithorst and Wilke, 2002; Imfeld et al., 2009). Although the neurobiology of structural plasticity in the brain remains unclear, the negative relationship between the volume of certain brain regions and speech and music performance may reflect more efficient neural organization caused by extensive experience. In light of this point, the reduced GM volume in the insula of the singers observed in the present study may reflect a refined neural network shaped by vocal training that allows for increased efficiency in the online detection and correction of errors in vocal output.

More importantly, we found that lower regional GM volumes in the bilateral insula were predictive of smaller vocal compensations for pitch perturbations. This finding is in line with the previously observed activation of insula in non-singers during the production of the early involuntary vocal compensations for pitch or F1 feedback perturbations during speaking (Toyomura et al., 2007; Tourville et al., 2008; Parkinson et al., 2012; Behroozmand et al., 2015). As well, singers and non-singers exhibited increased activity in the insula when they were instructed to voluntarily ignore or compensate for pitch feedback perturbations during singing (Zarate and Zatorre, 2008; Zarate et al., 2010). Thus, our results not only confirm and extend previous findings that the insula plays a special role in the online control of speaking and singing but also provide evidence for linking morphometric differences in insula between singers and non-singers to their distinct behavioral performance in auditory feedback control of vocal production. In light of the lesion findings that showed abnormally increased vocal compensations in patients with Parkinson’s disease, Alzheimer’s disease, and cerebellar degeneration as a result of an overreliance on auditory feedback (Chen et al., 2013; Huang et al., 2016; Parrell et al., 2017; Ranasinghe et al., 2017), the observed association between lower GM volumes in the insula and smaller vocal compensations suggests that extensive vocal training may refine the insula-based networks to weigh less heavily on auditory feedback (see more details below), facilitating the precise control of vocal pitch production.

### Neural Mechanisms of Speech Motor Control in Singers

While most studies have focused on the neurobehavioral correlates of vocal pitch regulation during singing in singers (Burnett and Larson, 2002; Zarate and Zatorre, 2005, 2008; Jones and Keough, 2008; Keough and Jones, 2009; Keough et al., 2013), the present study examined the experience-dependent modulation of auditory–vocal integration using a speaking task. Despite the methodological differences across these studies, a consistent finding is the significantly lower degree to which singers compensate for pitch perturbations in voice auditory feedback relative to non-singers. Moreover, neuroimaging studies have shown a largely overlapping neural network that supports auditory–vocal integration during singing (Zarate and Zatorre, 2005, 2008; Zarate et al., 2010) and speaking (Tourville et al., 2008; Parkinson et al., 2012; Chang et al., 2013; Behroozmand et al., 2015, 2017; Guo et al., 2016). Therefore, both speaking and singing studies point to experience-dependent mechanisms that support sensorimotor control of vocal production.

A plausible explanation is that singers may rely less on auditory feedback and more on somatosensory feedback during vocal pitch regulation. According to the DIVA model (Golfinopoulos et al., 2010), auditory feedback and somatosensory feedback are closely correlated and tightly integrated for fine-tuning of vocal motor production. Although how these two types of feedback are integrated is currently unclear, a growing body of literature has focused on the role of somatosensory feedback in speech motor control. For example, an increased reliance on auditory feedback is observed when somatosensory feedback is absent, as evidenced by significantly increased vocal compensations for pitch perturbations after anesthetizing the vocal folds (Larson et al., 2008). Some individuals are even able to adapt to changes in voice auditory feedback with a heavy reliance on somatosensory feedback, as reflected by the finding that participants who failed to adapt to auditory perturbations adapted to somatosensory perturbations when both auditory and somatosensory perturbations were presented simultaneously (Lametti et al., 2012). These findings suggest that a dynamic balance may exist between auditory and somatosensory feedback and when one form of feedback is compromised the speech motor system can compensate by using information from the other. In the context of singing, accumulating evidence has suggested that singers may weight somatosensory feedback more heavily for precise control of song production. For example, opera singers possessed increased GM volume in right S1 and S2 (Kleber et al., 2016) and exhibited increased activation in bilateral S1 during singing (Kleber et al., 2010). When auditory feedback was masked by noise, singers were still able to maintain pitch matching accuracy and showed increased activation in right anterior insula and its connectivity with SMG, whereas non-singers showed reduced pitch matching accuracy and decreased activity in right anterior insula and its connectivity with sensorimotor regions (Kleber et al., 2017). Therefore, it is possible that singers may develop a stronger reliance on somatosensory feedback to facilitate kinesthetic motor control of the vocal tract for the production of speech and song, thereby they are more capable of ignoring perturbations in auditory feedback and compensating for pitch perturbations to a lesser degree than non-singers.

In addition to feedback control, feedforward control also plays a critical role in speech motor control (Golfinopoulos et al., 2010) by enabling speakers to produce speech targets using previously learned motor commands without reliance on sensory feedback. There is evidence suggesting that singing expertise may lead to enhanced feedforward models that allow for precise control of vocal motor behaviors (Jones and Keough, 2008; Kleber et al., 2013). For example, Kleber et al. (2013) found that anesthesia of the vocal tract reduced pitch matching accuracy in singers to a lesser degree compared to non-singers, and this difference was accompanied by distinct pattern of brain activity under anesthesia across the two groups. Functional connectivity between right anterior insula, S1, A1, and M1 increased in non-singers, suggesting their recruitment of both the auditory and somatosensory network to produce the pitch targets. In contrast, singers exhibited decreased functional connectivity between the same areas and decreased activity in right anterior insula that was predictive of greater success in maintaining pitch matching accuracy under anesthesia (Kleber et al., 2013). This pattern of results suggests that the lack of somatosensory feedback did not lead to an increased reliance on auditory feedback; rather, singers may have largely relied on feedforward control developed during their vocal training that allowed them to ignore sensory feedback while maintaining pitch matching accuracy. This hypothesis is supported by one sensorimotor adaptation study by Jones and Keough (2008) that found higher voice *F*_0_ values during the aftereffects phase than those during the baseline phase for singers but not for non-singers. As an alternative explanation, therefore, singers may rely more heavily on feedforward control mechanisms but less on sensory feedback as a consequence of singing experience to produce the desired vocal targets, which results in decreased vocal compensations for pitch perturbations in auditory feedback accordingly.

### Limitations

Several limitations of the present study should be acknowledged. One primary limitation is that the use of the AAL template restricted our ability to link vocal pitch regulation to insula morphology at the subregional level. The insula can be functionally divided into three subdivisions: the posterior, ventral anterior, and dorsal anterior insula (Deen et al., 2011; Uddin et al., 2014). Using probabilistic diffusion tractography, Battistella et al. (2018) revealed distinct subdivisions of the insula and parallel, largely non-overlapping WM pathways with cortical regions involved in different motor aspects of speech production ranging from articulatory modulations to communicative motivation. It is possible that different insula subregions may be differentially influenced by singing experience that may lead to different structural changes (e.g., increased or decreased GM volumes). Future studies should be conducted to examine the contributions of different insula subdivisions to the different patterns of vocal pitch regulation observed between singers and non-singers. Another limitation of the present study is that only female singers without AP were recruited. Previous studies have demonstrated sex and AP effects on brain morphology in musicians (Hutchinson et al., 2003; Lee et al., 2003; Bermudez et al., 2009). There is also evidence that shows that the behavioral and neural processing of vocal pitch regulation varies as a function of sex (Chen et al., 2010; Swink and Stuart, 2012; Li et al., 2018) and AP (Behroozmand et al., 2014). Therefore, our results may not generalize across musician populations of both sexes or to individuals with AP. Finally, it has been documented that auditory feedback control of speech production varies as a function of language experience (e.g., Mandarin, Cantonese, English) (Liu et al., 2010; Chen et al., 2012; Ning et al., 2014, 2015). Therefore, we cannot also rule out the possibility that the relationship between insula morphology and vocal pitch regulation differs across different languages.

## Conclusion

The present study investigated the association between structural plasticity in insula as a function of singing experience and auditory-motor integration during vocal pitch regulation. Singers showed reduced regional GM volumes in the bilateral insula and decreased involuntary vocal compensations for pitch perturbations than non-singers, and smaller regional GM volumes in insula were significantly correlated with the magnitudes of vocal compensations. It is suggested that differential feedback and feedforward mechanisms may underlie the distinct pattern of speech motor control between singers and non-singers, which may be related to morphometric changes in insula as a result of singing training.

## Ethics Statement

All participants gave written informed consent in compliance with a research protocol approved by the Institutional Review Board of The First Affiliated Hospital at Sun Yat-sen University of China.

## Author Contributions

WW, GG, and HL designed the experiments. WW, LW, and NC performed the experiments and analyzed the data. WW, JJ, GG, and HL interpreted the results and wrote the manuscript. All authors read and approved the final manuscript.

## Conflict of Interest Statement

The authors declare that the research was conducted in the absence of any commercial or financial relationships that could be construed as a potential conflict of interest.

## Footnotes

1 http://www.fil.ion.ucl.ac.uk/spm/software/spm12/

2 http://dbm.neuro.uni-jena.de/cat/
